# Alzheimer’s Polygenic Risk Scores Associate with Atherogenic Lipids and Reduced CRP in Mid‐Life Women

**DOI:** 10.1002/alz70861_108460

**Published:** 2025-12-23

**Authors:** Judith Rose Harrison, Emily A Baker, Peter Holmans, Valentina Escott‐Price, Xavier Caseras, Evie Stergiakouli

**Affiliations:** ^1^ Translational and Clinical Research Institute, Faculty of Medical Sciences, Newcastle University, Newcastle upon Tyne, England UK; ^2^ Cardiff University, Cardiff, Wales UK; ^3^ Division of Psychological Medicine and Clinical Neurosciences, School of Medicine, Cardiff University, Cardiff UK; ^4^ UK Dementia Research Institute, Cardiff University, Cardiff UK; ^5^ MRC Integartive Epidemiology Unit (IEU) at the University of Bristol, Bristol UK

## Abstract

**Background:**

Metabolic and inflammatory dysregulation are increasingly implicated in Alzheimer’s disease (AD) aetiology. Genome‐wide association studies link AD risk loci to lipid metabolism and innate‐immune pathways, yet it remains unclear how polygenic burden manifests in peripheral biomarkers. We tested whether genome‐wide and pathway‐specific polygenic risk scores (PRS) for late‐onset AD are associated with circulating lipids and C‐reactive protein (CRP) in mid‐life adults.

**Method:**

Data came from 2,776 female participants (mean ± SD age = 48.0 ± 4.3 y) in the Avon Longitudinal Study of Parents and Children who provided fasting blood samples and genome‐wide genotypes. PRS were constructed from the largest GWAS of clinical AD cases to date (Kunkle 2019) using clumping (r² < 0.1, 500 kb) and a primary *p* ‐value threshold of 0.001. Scores were generated (i) genome‐wide, (ii) confined to eight biologically curated AD pathways, and (iii) with and without *APOE*‐region variants. Hierarchical linear regressions examined associations with LDL‐C, VLDL‐C, total cholesterol, HDL‐C and CRP, adjusting for age and ten ancestry principal components. False‐discovery‐rate correction controlled for multiple testing of traits and pathways.

**Result:**

Higher genome‐wide AD PRS predicted greater LDL‐C (p = 0.014) and total cholesterol (p = 0.044) but lower CRP (p = 1.2 × 10⁻⁴). Positive associations for LDL‐C and total cholesterol were also seen across seven lipid‐ and protein‐handling pathway PRS (p range = 6 × 100.03). CRP showed consistent negative associations with most pathway scores. All the reported associations survived FDR correction but became non‐significant when *APOE* variants were removed. Nevertheless, the trait variance explained by pathway PRS was often greater than that of *APOE* variants alone, indicating additional contributory loci outside *APOE*.

**Conclusion:**

In middle‐aged women, elevated polygenic liability for AD is linked to an atherogenic lipid profile and lower CRP concentrations, largely driven by *APOE* but with detectable contributions from wider AD‐associated pathways. These findings reinforce mechanistic ties between AD genetics, cholesterol homeostasis and systemic inflammation, and suggest that peripheral lipids could serve as quantifiable readouts of polygenic AD risk. Larger, longitudinal and multi‐omic studies are needed to clarify causal pathways and to determine whether non‐*APOE* genetic signals offer independent targets for dementia prevention.

